# Emotion Goals in Music Performance Anxiety

**DOI:** 10.3389/fpsyg.2020.01138

**Published:** 2020-06-15

**Authors:** Margaret S. Osborne, Brendan Munzel, Katharine H. Greenaway

**Affiliations:** ^1^Melbourne School of Psychological Sciences, The University of Melbourne, Melbourne, VIC, Australia; ^2^Melbourne Conservatorium of Music, The University of Melbourne, Melbourne, VIC, Australia

**Keywords:** emotion goals, emotion regulation, performance anxiety, musicians, performance

## Abstract

Performance anxiety can be debilitating, and so researchers and laypeople alike tend to assume that it is desirable to downregulate this emotion. Yet emerging perspectives in the emotion literature suggest that people sometimes aim to *upregulate* anxiety to aid performance. The present research investigated the emotion goals that musicians hold when performing. Drawing on a novel framework of emotion goals, the findings suggest that how people *want to feel* and how they want to *appear to feel* are determinants of performance anxiety. In Study 1 (*N* = 44), musicians mostly reported wanting to neither feel nor show anxiety during a performance, although a meaningful subset reported wanting to feel but not show anxiety during a performance. In Study 2 (*N* = 32), musicians who enacted an emotion goal to neither feel nor show anxiety reported less state unease and greater satisfaction with their performance than musicians who enacted a goal to feel but not show anxiety. This research yields insight into the emotion goals that musicians hold and how these goals influence desired performance outcomes.

## Introduction

Describing his experience of performance anxiety, 19th-century composer Frederic Chopin mused, “I feel choked by its breath, paralyzed by its curious glances, struck dumb by all those strange faces” (Jachimecki, 1937, p. 428). This perspective gels with broader evidence that anxiety is experienced as aversive, unpleasant, and uncontrollable (Gray, 1991) and that it manifests in behavioral cues such as vocal changes, prototypical facial expressions, and tremulousness that can impede effective action (Perkins et al., 2012). With such negative consequences, it is easy to assume that people would always wish to downregulate anxiety, particularly when it inhibits their ability to perform in desired ways. Yet emerging perspectives from the emotion literature suggest that people sometimes wish to feel negative emotions to meet higher-order goals (Tamir, 2016). The present research explores this idea in the context of music performance anxiety (hereafter referred to as MPA), investigating whether musicians aim to experience and express anxiety in strategic ways in order to aid performance, and the impact of these goals on actual performance.

### Understanding Emotion Goals

A burgeoning area of research in the emotion literature addresses people’s goals for their emotional state (Tamir, 2009). This question is separate from what emotions people *do* feel and rather seeks to understand what emotions people *want* to feel (Mauss and Tamir, 2014). In general, people hold hedonically focused goals to feel good and avoid feeling bad (Higgins, 1997; Larsen, 2000; Riediger et al., 2009). For example, a hedonically motivated pianist might aim to reduce anxiety because it feels subjectively unpleasant.

However, sometimes people hold instrumentally focused goals (Tamir, 2009) to feel more negative emotion in order to increase the benefits or reduce the costs associated with those emotional responses. Expectancy-value theory (Atkinson, 1957) provides an interpretation for these motives. The expected outcome of an emotion directly influences goals to experience the emotion (Tamir et al., 2015). For example, an instrumentally motivated trumpeter may expect that anxious arousal, although experienced as unpleasant, will improve performance and so have a goal to feel more anxiety.

Much of the research to date on emotion goals shows that intuitions about how emotion experience impacts action are important for how people want to feel in performance contexts. However, what has been missing from this literature is a consideration of the goals people hold for emotion expression in addition to experience. In other words, people do not only consider how they want to feel; they also routinely consider how they want to appear to feel to others. This may be particularly important in performance contexts, where people must focus not only on the quality of their own performance but also on how that performance is being received by an audience.

A new conceptualization of emotion goals takes both emotion experience and emotion expression into account (Greenaway and Kalokerinos, 2019). This paradigm separates unidimensional goals for emotion experience (e.g., to feel less anxious) and expression (e.g., to show less anxiety) and makes the case that emotion goals can in fact be bidimensional, such that different combinations of desire for experience and expression are possible (see Table 2 for a visual representation of the model). These combinations may involve goals for emotion experience and expression that are aligned or misaligned. That is, people may have a goal to experience and express emotion (Goal 1), to experience but not express emotion (Goal 2), to express but not experience emotion (Goal 3), or to neither experience nor express emotion (Goal 4).

Some of these combinations of goals may appear more unusual than others. Although it is relatively easy to find examples of emotion goals in which experience and expression track together—as in the case of wanting to feel and show positive emotion (Diener, 2000; Matsumoto et al., 2005) or neither feel nor show negative emotion (Egloff et al., 2006)—it is less intuitive to think of cases in which goals for experience and expression are misaligned. Nevertheless, these situations do occur.

In line with Goal 2 to experience but not express emotion, there are situations in which a person may wish to feel an emotion while not showing it to others. For example, winners sometimes spontaneously inhibit the expression of positive emotion (Friedman and Miller-Herringer, 1991; Kalokerinos et al., 2014; Schall et al., 2016) while aiming to savor the positive feeling that goes along with winning (Bryant et al., 2011). More relevant to the present context, people may choose to preserve an experience of anxiety to increase motivation and performance (e.g., Lane et al., 2011), while also aiming to project a calm exterior that hides this inner turmoil (Erber et al., 1996).

In the case of Goal 3 to express but not experience emotion, the literature on emotional labor suggests that this goal may be active when people have a social objective that is at odds with their internal state. Research on emotional labor describes this as “surface acting” (Grandey, 2000), which is generally studied in relation to the amplification of non-genuine positive emotions (e.g., “service with a smile”). Further evidence suggests that people in these situations do not always experience the emotion they express, which is referred to as “faking in bad faith” (Rafaeli and Sutton, 1987). Occupational examples of these phenomena include actors, customer service officers, or bill collectors who sometimes aim to express anger in order to motivate clients to pay but who do not necessarily report wanting to feel that emotion (Sutton, 1991; Grandey, 2000).

There is evidence to suggest that people hold bidimensional emotion goals at least as often as they hold unidimensional emotion goals and that certain bidimensional goals tend to be more common than others (Greenaway and Kalokerinos, 2019). For example, in a survey of 800 people, Greenaway and Kalokerinos (2019) found that almost all surveyed individuals (95%) could recall holding Goal 1 to experience and express emotion (regardless of whether that emotion was positive or negative). The least regularly reported bidimensional goal, although still highly common (86%), was Goal 4 to neither experience nor express emotion (again, for both positive and negative emotions). Goal 2 (88%) and Goal 3 (87%) fell within this range.

This early research shows that people do indeed have goals to influence how they feel and how they appear to feel. What is currently unknown is how these goals differ across contexts. In particular, very little is known about the emotion goals that people hold in performance contexts in general and in music performance specifically.

### Anxiety in Music Performance Contexts

Musical performance paradigms have been reliably used to induce state anxiety (Brooks, 2014). MPA is often described by musicians as aversive (Kenny, 2011) and is relatively common, with up to 70% of professional musicians reporting experiencing anxiety across performance contexts (Kenny, 2011). A meaningful 15–20% of performers exhibit debilitating MPA with considerable consequences for their personal and professional lives (Kenny et al., 2014), many of whom meet the criteria for a diagnosis of social anxiety disorder (American Psychiatric Association, 2013). Anxiety is often perceived as detrimental to music performance (Mor et al., 1995), but actual evidence that anxiety impedes performance is less clear-cut.

The functional consequences of anxiety are of considerable interest to performance disciplines (Beilock and Carr, 2001). The relationship between state anxiety and skilled task performance is classically described by an “inverted-U” function (Yerkes and Dodson, 1908). Moderate anxiety may increase motivation and behavior tendencies for action (Eysenck et al., 2007), while high-intensity anxiety produces worry, escape, and avoidance tendencies (Frijda, 1986). These observations hold clues to the emotion goals that people may hold for anxiety, particularly in performance contexts.

The emotion literature in general shows mixed evidence for whether people aim to increase or decrease the experience of anxiety. In general, people appear to want to experience less anxiety (Tamir et al., 2007; Tamir and Ford, 2012), but there are specific cases in which people want to feel more anxiety, and these cases often occur in performance contexts. For example, Lane et al. (2011) reported that 15% of runners believe that anxiety facilitates athletic performance. The belief that anxiety is useful has also been seen in other performance contexts, such as experimental tasks with financial rewards (Tamir et al., 2015). This suggests that when anxiety experience is expected to benefit performance in a given context, people will aim to increase it.

There is less evidence concerning people’s goals for increasing or decreasing the expression of anxiety, although some exists (e.g., Egloff et al., 2006; Johns et al., 2008). In an observational study, Egloff et al. (2006) measured anxiety regulation in an evaluated public speaking task and found that participants used regulation strategies to decrease the appearance of anxiety in this context (Egloff et al., 2006). A similar finding was reported in a quasi-experimental study using a test-anxiety paradigm by Johns et al. (2008). Generally speaking, then, people appear to want to decrease the expression of anxiety, although they are more mixed on whether they aim to decrease or increase the experience of anxiety. This suggests that Goal 4 (neither experience nor express anxiety) and Goal 2 (experience but not express anxiety) may be particularly commonly held in performance contexts.

### The Present Research

After the literatures on MPA and emotion goals were reviewed, it is unclear what emotion goals musicians typically hold in performance settings. On the one hand, anxiety sometimes impedes performance and its expression may be perceived negatively by an audience. On the other hand, anxiety sometimes improves performance and its expression may sometimes inspire compassion in an audience. Thus, it is plausible that musicians may hold one of several bidimensional emotion goals. For example, to improve performance and elicit support, some musicians may aim to both experience and express anxiety (Goal 1). Others may aim to get a performance boost from experiencing anxiety but simultaneously aim to appear cool and collected by not expressing anxiety (Goal 2). For strategic reasons, some musicians may aim to express anxiety to garner sympathy but to not experience the anxiety to smooth their performance (Goal 3), whereas others may aim to eliminate anxiety from their performance entirely by neither experiencing nor expressing it (Goal 4).

Because no research has investigated which emotion goals musicians hold, it is unclear which of the above goals will be more common. The present research is the first to directly ask musicians how they want to feel and how they want to appear to feel during music performances. To elicit base rate information, Study 1 asked musicians which emotion goals they could recall holding during past performances. To understand the impact of emotion goals on relevant outcomes, Study 2 experimentally manipulated the two most frequently reported goals musicians held from Study 1 and assessed the impact on state anxiety and self-rated performance quality. In all, the studies provide a novel window into how musicians use their emotions strategically to guide performance, and whether their instincts about how emotion should be used result in beneficial outcomes.

## Study 1

Study 1 was an exploratory study to examine the frequency of bidimensional emotion goals in music performance situations. Individuals were asked whether they could remember holding each of four emotion goals: to experience and express anxiety (Goal 1), to experience but not express anxiety (Goal 2), to express but not experience anxiety (Goal 3), and to neither experience nor express anxiety (Goal 4). MPA and trait anxiety were measured in order to track potential relationships with specific emotion goals.

### Method

#### Participants

A convenience sample of 44 musicians (*M*_age_ = 24.70 years, *SD* = 9.91) participated in Study 1. Eligibility criteria included being 18 years or older and having at least 5 years’ experience with the main musical instrument (*M*_years_ = 12.67 years, *SD* = 6.56). After approval from the researchers’ university human research ethics committee was obtained, participants were recruited by email using music faculty listings, Facebook advertising, or advertising during university concert classes. Demographics and performance characteristics for both studies are reported in Table 1. Study 1 participants comprised predominately female undergraduate musicians specializing in piano. Around 15% of participants reported prior psychotherapy or pharmacotherapy for severe performance anxiety, and two participants reported both.

**TABLE 1:** Demographics across the studies.

	**Study 1**		**Study 2**	
	***n***	**%**	***n***	**%**
**Gender**				
Male	12	27.3	9	28.1
Female	32	72.7	23	71.9
Total	44	100	32	100
**Principal musical instrument**				
Keyboard	16	36.4	15	46.9
Guitar	4	9.1	2	6.3
String	5	11.4	3	9.4
Woodwind	5	11.4	4	12.5
Brass	4	9.1	3	9.4
Voice	8	18.2	4	12.5
Other	2	4.5	1	3.1
Total	44	100	32	100
**Musical role**				
Undergraduate	32	72.7	23	71.9
Postgraduate	9	20.5	4	12.5
Performing musician	3	6.8	3	9.4
Total	44	100	30	93.8
**Psychotherapy for MPA**				
Yes	7	15.9	5	15.6
No	37	84.1	25	78.1
Total	44	100	30	93.8
**Pharmacotherapy for MPA**				
Yes	5	11.4	5	15.6
No	39	88.6	25	78.1
Total	44	100	30	93.8

*MPA, music performance anxiety.*

#### Measures

##### Demographics

Demographic information was gathered on age and gender, performer-relevant information concerning main instrument, musical role, years of experience, and previous psychotherapy or pharmacotherapy for MPA.

##### Emotion goal

Participants were asked about four specific types of emotion goals that they may have felt during music performance, which were adapted from Greenaway and Kalokerinos (2019). The goals are outlined in Table 2. Participants responded to the question “Do you recall ever holding this goal for anxiety, specifically when performing music?” with response options of yes or no.

**TABLE 2:** Emotion goal measures in Study 1.

	**Expression**	
	**Increase anxiety**	**Decrease anxiety**
**Experience**		
Increase anxiety	***GOAL 1***	***GOAL 2***
	***Experience and Express Anxiety***	***Experience but not Express Anxiety***
	Please think about a performance situation in which you have actively wanted to BOTH **experience** AND **express** anxiety. This is a situation in which you deliberately wanted to feel anxious, and deliberately express your anxiety.	Please think about a performance situation in which you have actively wanted to **experience** BUT NOT **express** anxiety. This is a situation in which you deliberately wanted to feel anxious, BUT deliberately NOT express your anxiety.
Decrease anxiety	***GOAL 3***	***GOAL 4***
	***Express but not Experience Anxiety***	***Neither Experience nor Express Anxiety***
	Please think about a performance situation in which you have actively wanted to **express** BUT NOT **experience** anxiety. This is a situation in which you deliberately wanted to show anxiety, BUT deliberately NOT experience or feel your anxiety.	Please think about a performance situation in which you have actively wanted to NEITHER **experience** NOR **express** anxiety. This is a situation in which you deliberately wanted to NOT feel anxious, and NOT show your anxiety.

*Experience = the emotion a person feels. Express = the emotion a person appears to feel.*

##### Music performance anxiety

The Kenny Music Performance Anxiety Inventory (KMPAI; Kenny, 2011) was used to measure trait MPA severity. This measure operationalizes a prominent model of MPA (Kenny, 2005b). The KMPAI measures previous anxiety severity and psychological vulnerabilities associated with developing debilitating MPA (Barlow, 2002). The scale measures agreement with 40 items (eight items reverse coded) assessed on a seven-point Likert scale anchored from 0 (strongly disagree) to 6 (strongly agree). Reliability was good (Guttman’s coefficient λ = 0.87). Higher scores indicate debilitating MPA, with some authors advocating a diagnostic cutoff score of 105 for severe and maladaptive MPA (Ackermann et al., 2014).

##### Trait anxiety

The State-Trait Anxiety Inventory trait subscale (STAI-T; Spielberger, 1983) measures relatively stable individual differences in the tendency to worry, feel anxious, and perceive stressful situations as threatening. Twenty items (nine reverse scored) assess these differences, each on a four-point Likert scale anchored from 1 (almost never) to 4 (almost always). Reliability was good (Guttman’s coefficient λ = 0.92). Higher total scores indicate higher levels of trait anxiety.

### Results

#### Preliminary Analyses

Missing data were identified for dependent variables on 5% of cases and on baseline psychometric measures on 12% of cases. Analysis suggested that data were missing completely at random, Little’s test *χ*^2^(2) = 1.01, *p* = 0.604, before being pairwise deleted. Indices of non-normality of standardized residuals for continuous variables suggest that this assumption was met for all variables other than age. Univariate outlier analysis did not identify any extreme data points outside the range of ±3.29 standard deviations (Tabachnick et al., 2007). On average, this sample represented a group with very high trait MPA (*M*_KMPAI_ = 120.90) and high trait anxiety (*M*_STAI–T_ = 46.50) as compared with similar musician samples (Kenny et al., 2014).

#### Emotion Goal Frequency

As seen in Table 3, the majority of participants (83%) identified previously holding Goal 4 (neither experience nor express anxiety) in past performances. The second most frequently reported was Goal 2 (experience but not express anxiety) with 30% of participants previously holding this goal. Goal 1 (experience and express anxiety) and Goal 3 (express but not experience anxiety) were less commonly reported.

**TABLE 3:** Frequency of bidimensional goals with respect to music performance.

	**Study 1**				**Study 2**	
	**Yes**		**No**		**Selected**	
	***n***	**%**	***n***	**%**	***n***	**%**
Goal 1	12	27.3	32	72.7	8	25.0
Goal 2	13	30.2	30	69.8	10	31.3
Goal 3	11	26.2	31	73.8	3	9.4
Goal 4	35	83.3	7	16.7	11	34.4

*Participants in Study 1 rated whether they could recall ever holding (or not) a specific emotion goal (more than one goal could be selected) in a past performance. Participants in Study 2 rated only one goal that they were specifically adopting for the upcoming trial performance. *n*, frequency count; *Goal 1*, to experience and express anxiety; *Goal 2*, to experience but not express anxiety; *Goal 3*, to express but not experience anxiety; *Goal 4*, neither experience or express anxiety.*

It was not the case that performance anxiety (KMPAI) or trait anxiety (STAI-T) tracked with holding specific emotion goals. For example, people who reported having held Goal 4 (neither experience nor express anxiety) scored no higher on the KMPAI, *t*(37) = 0.77, *p* = 0.448, or STAI-T, *t*(37) = −1.18, *p* = 0.861, than people who reported never having held Goal 4. Likewise, people who reported having held Goal 2 (experience but not express anxiety) scored no higher on the KMPAI, *t*(37) = −0.08, *p* = 0.933, or STAI-T, *t*(37) = 1.47, *p* = 0.149, than people who reported never having held Goal 2.

## Study 2

Study 1 indicated that musicians are particularly likely to hold Goal 4—neither experience nor express anxiety—but that a subset also report holding Goal 2—experience but not express anxiety. Having held these goals was not associated with trait-like measures of performance anxiety or generalized anxiety, leaving open the possibility that these goals may be associated with state anxiety when adopted in specific performance contexts.

Study 2 focused on the impact of emotion goals on state anxiety using an experimental design with repeated-measures methodology. The study manipulated within subjects the two most frequently reported emotion goals nominated in Study 1: Goal 4 and Goal 2. Anxiety was induced using a music performance paradigm. The main outcome measures were state anxiety and self-rated performance quality.

### Method

#### Participants

An *a priori* power analysis was conducted using G^∗^Power, Version 3.1 (Faul et al., 2009). Based on conservative values of alpha (α = 0.05) and power (β = 0.80), this suggested that a sample size of 34 would be appropriate to detect a medium effect (*d* = 0.50).

Thirty-two participants (*M*_age_ = 22.90 years, *SD* = 6.75) from Study 1 completed the study using the same methods of recruitment and inclusion criteria. The sample comprised predominantly female undergraduate pianists (Table 1) with considerable musical experience (*M* = 12.90 years, *SD* = 4.20). Approval for the study was granted by the researchers’ university human research ethics committee, and each participant was compensated $10 for their participation.

#### Design

The study employed a one-way within-subjects design in which participants were encouraged to adopt Goal 2 (experience but not express anxiety) and Goal 4 (neither experience nor express anxiety) in a music performance. The order of goal adoption was counterbalanced across participants to control for order effect by block randomizing the participants (Suresh, 2011).

#### Measures

##### Baseline emotion goal

Participants were asked to report their emotion goal prior to completing the performance by responding to the item “Going into this performance, what is your goal in relation to influencing performance anxiety?” with four response options: *To BOTH experience AND express anxiety; To experience BUT NOT express anxiety; To express BUT NOT experience anxiety; To NEITHER experience NOR express anxiety* (Greenaway and Kalokerinos, 2019).

##### Goal efficacy

Participants were asked to rate their ability to enact the assigned emotion goal manipulation after each condition (“How effective were you in meeting your goal to NEITHER experience nor express anxiety?”; “How effective were you in meeting your goal to experience BUT NOT express anxiety?”). Responses were recorded on a scale ranging from 1 (not at all effective) to 7 (extremely effective).

##### State anxiety

State anxiety was measured using an adapted version of the *Competitive State Anxiety Inventory-2 (CSAI-2R*; Cox et al., 2003). The CSAI-2R measures precompetitive state anxiety in cognitive and somatic domains, and self-confidence. CSAI-2R comprises 17 items on a four-point Likert scale anchored from 1 (strongly disagree) to 4 (strongly agree). We adapted the CSAI-2R, using 12 items loading cognitive (items 2, 4, 6, 8, and 10) and somatic subscales (items 1, 3, 5, 7, 9, 11, and 12). Likert scales were expanded over nine points across the same anchors, in line with previous research (Osborne and McPherson, 2019). Subscale and total scores were calculated by dividing the total scores by the number of items.

##### Performance quality

Participants were asked to rate the quality of each performance (“Reviewing the quality of the performance you just gave, overall, to what extent did you perform this piece with complete musical and technical accuracy?”) on a scale ranging from 0 (not at all) to 100 (a great deal).

#### Procedure

The study used a music performance paradigm undertaken in musical rehearsal spaces of similar size, lighting, and acoustics located on familiar university campuses. Participants performing piano repertoire used an instrument supplied. All other participants performed using their own instrument. Demographics and other performance relevant measures were collected as in Study 1.

Participants provided written informed consent and were given an information sheet that contained laydefinitions of anxiety experience and expression domains, emotion goals, and regulatory strategies. Participants were allowed 5 min to “warm-up” on their main instrument. Measures taken at baseline included state anxiety and baseline emotion goal selection.

Goal manipulation involved verbal instructions that were adapted from descriptions of emotion goals described by Greenaway and Kalokerinos (2019). Goal manipulations were read aloud by the student researcher while participants remained seated with their eyes closed, followed by a brief pause (30 s). Verbal goal instructions for manipulations with Goal 2 (experience but not express anxiety) and Goal 4 (neither experience nor express anxiety) are presented in Table 4.

**TABLE 4:** Verbal instructions for emotion goal prompts.

**Goal**	**Verbal instructions**
Goal 2	Now I would like you to deliberately feel anxious about the performance you are about to give. Build it up so you can really notice your nervous energy. You could do this by thinking about how you are going to play and what people might think about your performance. You could imagine your heart pounding in your chest, your heart rate and muscle tension increasing. Take some short quick shallow breaths and notice any sensations of butterflies in your stomach, shaking or trembling, dryness in your mouth, or sweaty hands. Now that you are experiencing some anxiety about the performance, I would like you to restrict your expression of anxiety. Please make a deliberate attempt to feel but not show any anxiety as you perform this piece. Try to behave in a way that would not give me or anyone who may watch this video of your performance any visible clues about the performance anxiety you are experiencing.
Goal 4	Now I would like you not to feel any anxiety about your performance. When you play this piece, you don’t want to experience any performance anxiety. It is also important that you not show any anxiety. Try to behave in a way that would not give me or anyone else who may watch this video of your performance any visible clues of anxiety. Please make a deliberate attempt not to feel or show any anxiety as you perform this piece

Following the goal manipulation, participants were verbally instructed to “Please prepare for the performance. When you are ready, let me know and we will begin recording.” Each participant performed a 3-min excerpt from a familiar piece of musical repertoire on their main instrument. Each performance was video and audio recorded. The student researcher remained present for each performance. At completion of the performance, participants were instructed to return to their seat and provide responses to the relevant measures.

Experimental measures were collected for self-reported ability to execute the goal manipulation, state anxiety, and self-reported performance quality. The experimental procedure was repeated for each participant under each goal. Goal order was counterbalanced. On completion of the study, participants were asked if they would like to provide any additional feedback and were debriefed.

### Results

#### Preliminary Analyses

There were no missing data for dependent variables. Univariate outlier analysis did not identify any extreme data points outside the range of ±3.29 standard deviations on core variables (Tabachnick et al., 2007).

#### Emotion Goal Frequency

As in Study 1, Goal 4 (neither experience nor express anxiety; 34%) and Goal 2 (experience but not express anxiety; 31%) were the most frequently endorsed types of emotion goals. The frequencies for all goals are displayed in Table 3.

#### Experimental Goal Effects

##### Goal efficacy

A one-way repeated-measures ANOVA was applied to perceived ability to enact the specified emotion goal (controlling for order of goal manipulation). There was no significant difference between the conditions, *F*(1,30) = 1.35, *p* = 0.254, η_*p*_^2^ = 0.04, indicating that there was no difference between perceived efficacy for Goal 2 (*M* = 4.34, *SD* = 1.52) and Goal 4 (*M* = 4.56, *SD* = 1.29).

##### State anxiety

**FIGURE 1:**
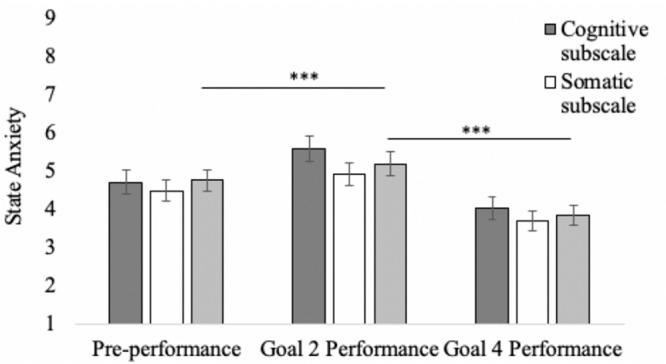
State anxiety (CSAI-2R total score, cognitive and somatic subscales) as a function of goal condition. *Goal* 2 is to experience but not express anxiety. *Goal* 4 is to neither experience or express anxiety. Significant differences denoted by ^∗∗∗^*p* < 0.001.

A one-way repeated-measures ANOVA was applied to state anxiety ratings collected before the performance and following each performance (controlling for order of goal manipulation). There was a significant effect, *F*(2,29) = 20.01, *p <* 0.001, η_*p*_^2^ = 0.58, such that state anxiety significantly increased from preperformance (*M* = 4.58, *SD* = 1.54) to postperformance after enacting Goal 2 (*M* = 5.20, *SD* = 1.67), *p* = 0.044, but anxiety significantly decreased from preperformance to postperformance after enacting Goal 4 (*M* = 3.84, *SD* = 1.45), *p* = 0.003. Anxiety was significantly higher after enacting Goal 2 compared with Goal 4, *p* < 0.001; see Figure 1. As seen in Figure 1, the pattern of results was extremely similar when the cognitive, *F*(2,29) = 18.41, *p <* 0.001, *η*_*p*_^2^ = 0.56, and somatic subscales, *F*(2,29) = 16.48, *p <* 0.001, *η*_*p*_^2^ = 0.53, were considered separately.

##### Performance quality

**FIGURE 2:**
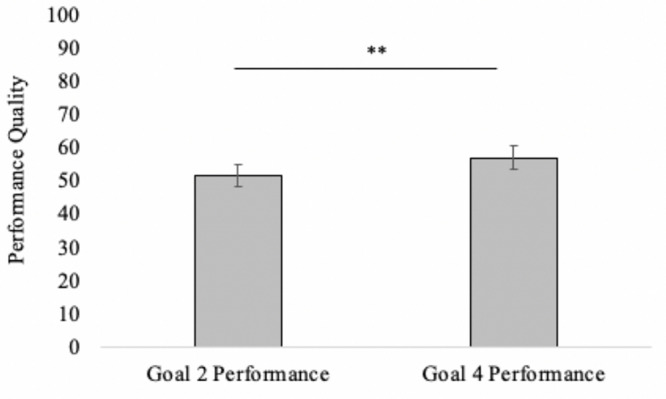
Self-rated performancequality as a function of goal condition. *Goal* 2 is to experience but not experess anxiety, *Goal* 4 is to neither experience or express anxiety. Significant differences denoted by ^∗∗^*p* = 0.011.

A one-way repeated-measures ANOVA was applied to self-rated performance quality (controlling for order of goal manipulation). There was a significant effect, *F*(1,30) = 7.36, *p* = 0.011, η_*p*_^2^ = 0.20, such that performance quality was rated as higher after enacting Goal 4 (*M* = 57.19, *SD* = 19.35) than Goal 2 (*M* = 51.53, *SD* = 18.66); see Figure 2.

##### Indirect effects

Bootstrapping analyses with 10,000 resamples were conducted to test the indirect effects of emotion goal on self-rated performance quality *via* state anxiety using the Montoya and Hayes (2017) *memore* macro for repeated-measures mediation. The indirect effect was significant (*IE* = −7.17, *SE* = 2.53, 95% CIs = −12.47 to −2.64), suggesting that emotion goals impacted performance satisfaction *via* changing people’s emotional state in goal congruent ways. That is, Goal 2 (experience but not express anxiety) increased anxiety and thus reduced self-rated performance quality, whereas Goal 4 (neither experience nor express anxiety) reduced anxiety and thus improved self-rated performance quality.

## General Discussion

This research provides the first insight into which emotion goals musicians hold when trying to regulate performance anxiety. Anxiety levels in our participants were relatively high, making them a useful sample in which to investigate this question. Together, the results suggest that the musicians we sampled had relatively uniform preferences for the emotion goal they held and that this emotion goal appeared to be useful in helping them perform well.

One retrospective study and one experiment found that our musicians were most likely to report holding an emotion goal to neither experience nor express anxiety (Goal 4). The next most common emotion goal to be reported was one to experience but not express anxiety (Goal 2). The overall preference for Goal 4 is consistent with literature suggesting that anxiety is aversive (Gray, 1991; Kenny, 2011). The next most common preference for Goal 2 is also consistent with evidence that although people vary in terms of whether they believe experiencing anxiety is good for performance, they appear relatively united in aiming not to show anxiety. This is consistent with literature showing that people often aim to appear “cool, calm, and collected” when performing for others (Erber et al., 1996; Egloff et al., 2006). Hence, both goals feature a desire to downregulate the expression of anxiety but vary on whether experiencing anxiety is desirable or not.

It is interesting to contrast this base rate with other samples. In a general community sample, Greenaway and Kalokerinos (2019) found that people on average were slightly less likely to recall holding Goal 4 (86% of the sample) than, for example, Goal 1 to experience and express emotion (95% of the sample). This was true for negative emotion as well as positive emotion. The flip of this prevalence, especially given the dramatic difference between Goal 4 (83% of the sample in Study 1) and Goal 1 (27% of the sample in Study 1), suggests that performance anxiety may be a specific case in which emotion goals are different from the norm.

Study 2 revealed the impact of holding the two most commonly identified emotion goals from Study 1 on state anxiety and self-rated performance quality. Counterbalancing the order of goal enactment, participants were instructed to hold an emotion goal to experience but not express anxiety (Goal 2) or to neither experience nor express anxiety (Goal 4) while performing a piece of music from their repertoire. The results revealed that, consistent with the emotion goal, Goal 2 increased the experience of anxiety for participants relative to preperformance baseline, whereas Goal 4 decreased the experience of anxiety. This was true for both cognitive and somatic channels of anxiety. Thus, performers appear capable of enacting instructed emotion goals, and doing so changes their emotion in goal-consistent ways.

Even more telling, self-rated performance quality changed with emotion goal adoption. Participants who enacted an emotion goal to experience but not express anxiety rated their performance as worse than participants who enacted an emotion goal to neither experience nor express anxiety. This finding begins to hint at which emotion goal might result in the best performance for those suffering from performance anxiety. It also suggests that the instincts of participants in Study 1 were well tuned—these participants reported being able to remember holding Goal 4 to neither experience nor express anxiety much more commonly than the other goals. The findings of Study 2 suggest that this may have resulted in a better self-perceived performance.

### Theoretical and Practical Implications

This research represents the first application of a bidimensional emotion goal model (Greenaway and Kalokerinos, 2019) in an applied context. In addition to advancing the ecological validity of this model, the present findings suggest that the emotion goals that people hold are sensitive to context, with performance contexts perhaps being more likely to inspire Goal 4 than other contexts. Future research should systematically investigate emotion goals in other situations to provide a taxonomy of how desire for emotion experience and expression shift in ways that are sensitive to context.

This research is also the first to show that musicians hold a range of emotion goals in relation to performance anxiety. Although researchers and practitioners may have assumed that musicians always want to downregulate anxiety, the present findings suggest this is not always the case. In fact, some people aim to experience anxiety while performing, presumably out of a belief that such anxiety is in fact adaptive. Our findings suggest that it does not appear particularly beneficial to experience anxiety while performing, although this may have been because participants were also aiming to simultaneously *not show* anxiety (i.e., were enacting Goal 2). A more useful approach may involve reappraising anxiety as excitement, which has been shown to fuel performance (Brooks, 2014).

Treatment of debilitating MPA can be challenging (Kenny, 2005a) and often relies on a mixture of approaches including behavioral strategies, pharmacotherapy, and psychotherapies (e.g., cognitive behavioral therapy and acceptance commitment therapy; Clarke et al., 2020). Advancing understanding of emotion goals in MPA may have implications for tailoring therapies. For example, many therapies assume that people want to downregulate anxiety in general, without considering the possibility that people may instead have a preference for downregulating the expression but not experience of anxiety, or vice versa. Effective regulation relies on an understanding of how people *want* to feel, as well as useful methods for changing one’s actual emotional state. One path forward for treating MPA may involve checking with musicians specifically about what their emotion goal is prior to suggesting methods of regulating anxiety.

### Limitations and Future Directions

Of course, like all studies, the ones performed in this research had a number of limitations. One wound to internal validity relates to the sampling method. We aimed to study normative anxiety regulation in a representative musician sample. However, the musician sample showed marked MPA and dispositional anxiety. Self-selection bias can alter a sample composition on the basis of individual difference variables like trait anxiety, relative to the corresponding parent population (Almeida et al., 2008). While convenience sampling is efficient and convenient, future research investigating normative emotion regulation in musicians may need to consider random sampling approaches to minimize this bias.

Another weakness in relation to construct validity concerns the outcome measure used to capture anxiety and performance. These variables were measured *via* self-report and for anxiety only in the domain of experience. Emotions are multifaceted phenomena occurring on multiple dimensions (Dan-Glauser and Gross, 2011). A more well-rounded representation of this construct could be achieved using additional physiological and behavioral indices to capture emotion over time (Dan-Glauser and Gross, 2013). Likewise, performance quality could be evaluated more objectively by an audience who is present at the time of the performance. This may lessen the effects of social-desirability and response-shift biases that are particularly problematic for self-report in experimental designs (Rosenman et al., 2011).

In terms of future directions, the present research investigated emotion goals in a context in which one goal tends to be primary—the goal to neither experience nor express anxiety. It is possible that the performance results observed in Study 2 are indicative of what happens when people are extrinsically encouraged to adopt an emotion goal that is not their natural preference. This is, if most of our participants in Study 2 wanted to neither experience nor express anxiety naturally but were asked to try to experience anxiety when enacting Goal 2, this may have resulted in worse outcomes than if these participants were left to freely enact their preferred goal. This might suggest that a regulatory fit (Higgins, 2005) results from wanting to hold a particular emotion goal and being free to enact that goal in performance settings.

### Conclusion

The present research offers insight into the goals that musicians hold for how much anxiety they want to feel and how much anxiety they want to be seen to feel in performance contexts. As such, this work contributes to the burgeoning literature on emotion goals, as well as helping to understand their impact on performance. Findings over two studies suggest that musicians hold goals to reduce anxiety experience and expression in music performance and that this is an adaptive goal to hold to the extent that it improves self-rated performance. In all, the findings lay the groundwork for suggesting it is first important to understand the emotion that people want to feel and show before suggesting methods of dealing with performance anxiety.

## Data Availability Statement

The datasets generated for this study will not be made publicly available. The small sample size may compromise anonymity. Requests to access the datasets should be directed to the corresponding author.

## Ethics Statement

The studies involving human participants were reviewed and approved by Human Research Ethics Committee, The University of Melbourne. The patients/participants provided their written informed consent to participate in this study.

## Author Contributions

MO and KG developed the study concept and designed the research. BM collected data and performed initial data analysis. KG prepared the manuscript. All authors edited, read and approved the final manuscript.

## Conflict of Interest

The authors declare that the research was conducted in the absence of any commercial or financial relationships that could be construed as a potential conflict of interest.
